# Supplementary material for: Comparative genomics reveals LINE-1 recombination with diverse RNAs
Source: Cell Genom. 2026 Feb 17;6(5):101165. doi: 10.1016/j.xgen.2026.101165 (PMC13174262; doi:10.1016/j.xgen.2026.101165)
Supplement: Document S1. Figures S1–S9 and Table S1 [file mmc1.pdf]

Cell Genomics, Volume *6*

Supplemental information

Comparative genomics reveals LINE-1 recombination with diverse RNAs

Cheuk-Ting Law and Kathleen H. Burns

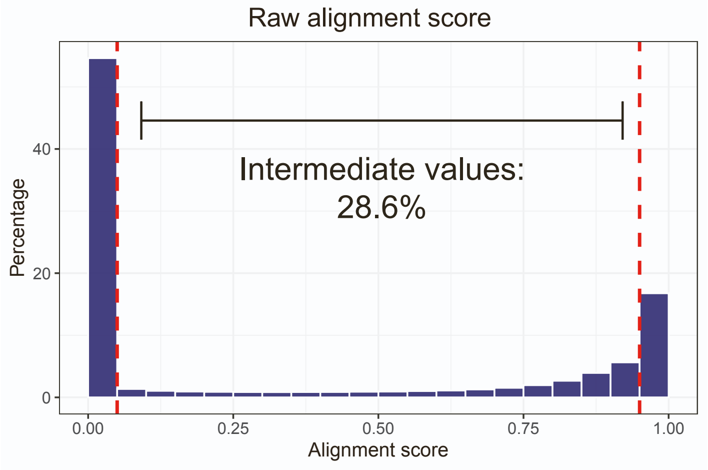

Figure S1. Distribution of LINE-1 alignment scores in multiple sequence alignment (MSA) across 470 mammalian genome assemblies, Related to Figure 1.

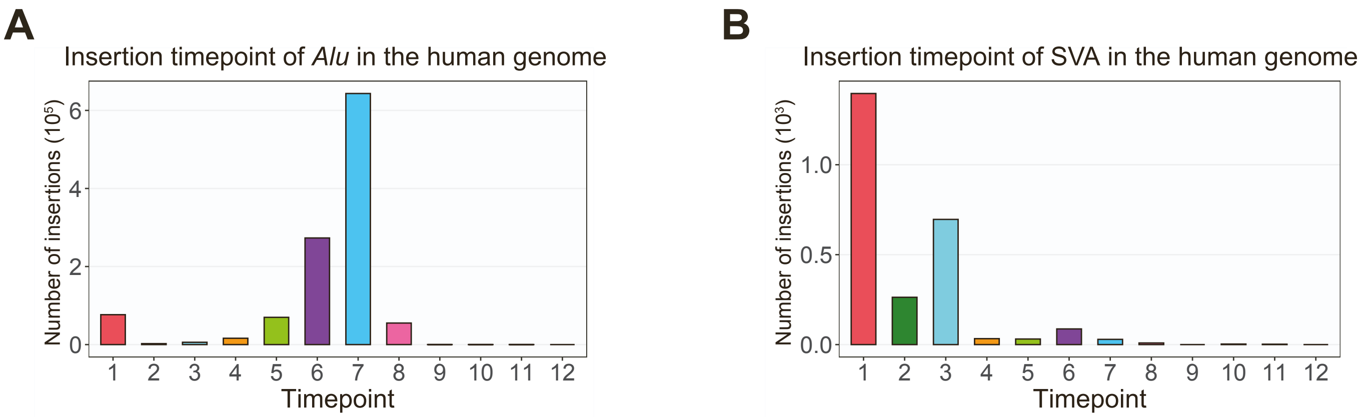

Figure S2. The frequency of insertion timepoint of selected TE family in the human genome assembly, Related to Figure 2. (A) The majority of *Alu* elements were inserted at timepoints 6 and 7, representing their peak activity. *Alu* retrotransposition declined significantly after timepoint 5, but a resurgence of activity is observed in the human genome, indicating a recent wave of *Alu* mobilization. (B) Over 90% of SVA insertions occurred between timepoints 1 and 3, with the highest activity observed in *Homo sapiens*.

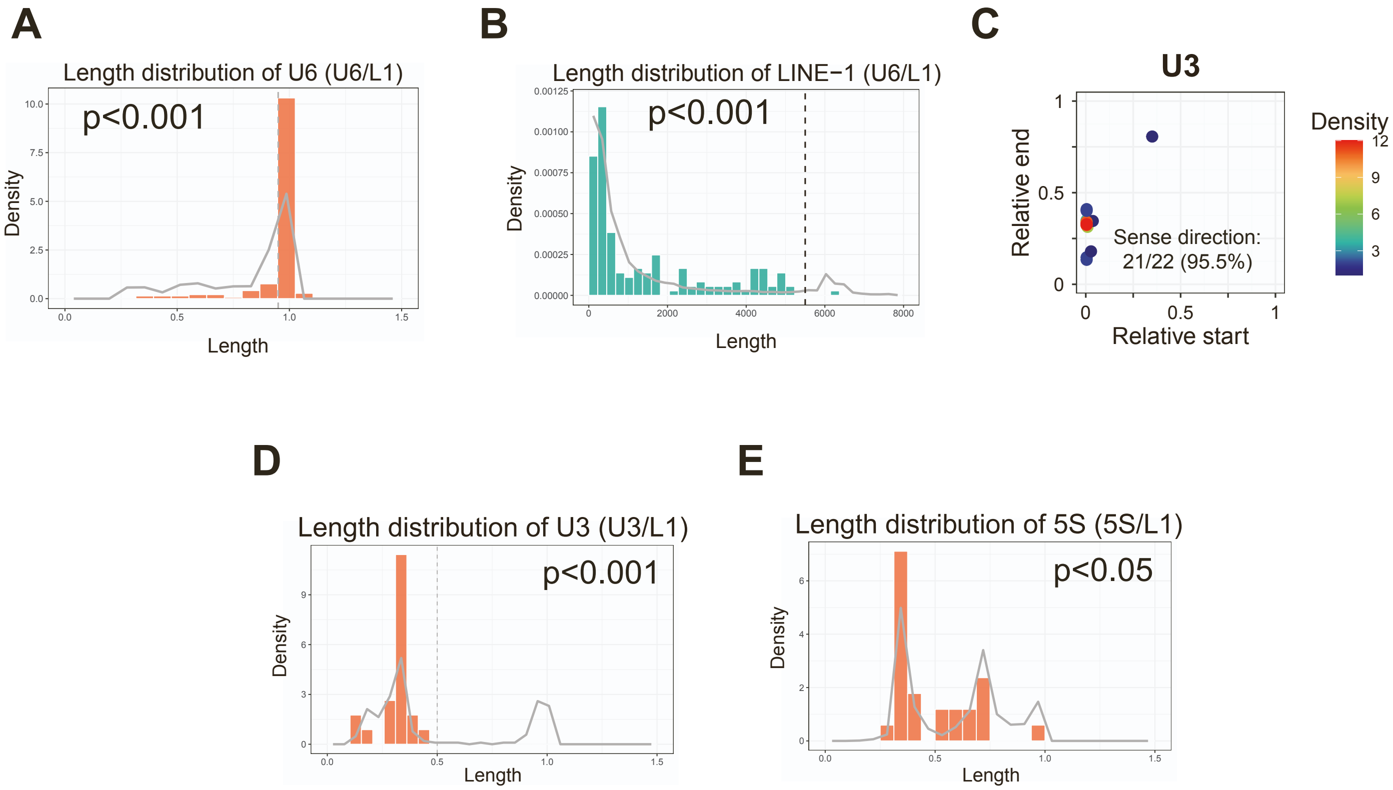

Figure S3. Characterization of RNA repeat associated LINE-1 chimeric insertions, Related to Figure 3. (A) U6 RNA sequences in U6/LINE-1 chimeras show a significantly higher proportion of full-length sequences (defined as ≥95% of the length of the consensus U6 sequence) compared to other genomic U6 RNA (indicated by the grey line), as determined by Fisher's exact test. (B) LINE-1 sequences in U6/LINE-1 chimeras are frequently truncated (length < 5,500 bp) compared to all genomic LINE-1 elements (L1PA and L1PB families). This association was statistically tested using Fisher's exact test. (C, D) Among the identified 22 U3/LINE-1 chimeric insertions, the U3 segment is frequently truncated, with length approximately 30% of the U3 sequence. This is notably shorter when compared to U3 sequences generally. This association was evaluated using Fisher's exact test, defining truncated events as <50% of the full-length U3. Most U3 sequences are oriented in the sense direction relative to their LINE-1 counterpart. (E) The length distribution of 5S rRNA in 5S/LINE-1 chimeric insertions differs significantly from those found independently of LINE-1. Wilcoxon Rank-Sum Test was used due to the non-bimodal distribution of 5S RNA lengths.

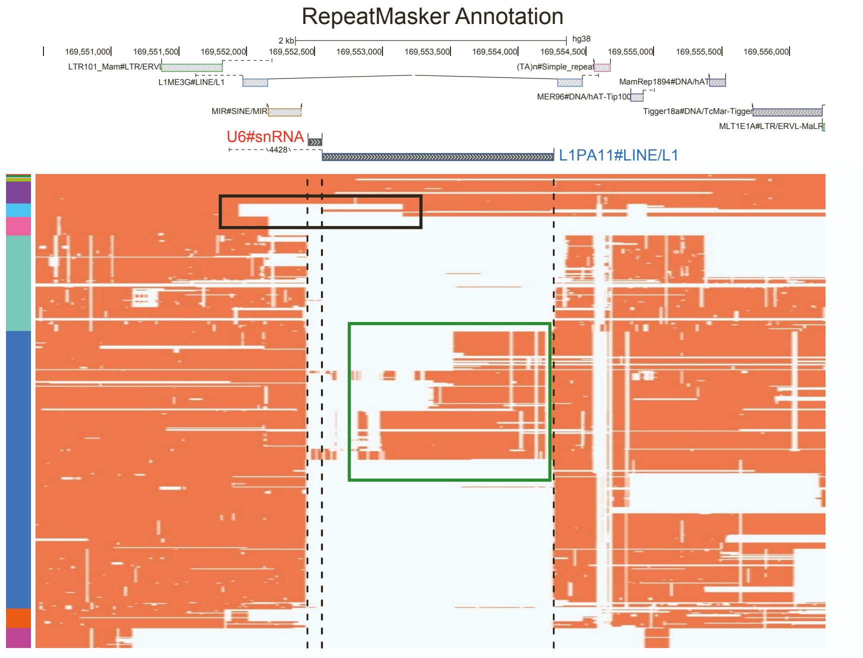

Black square: Regional deletion in New World Monkeys

Green square: Misalignment of similar LINE-1 sequences

Figure S4. An example of a true U6/LINE-1 chimeric insertion excluded by the computational pipeline, Related to Figure 3. The region containing the U6 sequence and part of LINE-1 (indicated by the black rectangle) is deleted in the clade of New World monkeys. This deletion leads to an inaccurate inference of the U6 insertion timing, resulting in exclusion of this locus due to its misclassification as a non-contemporaneous insertion. Additionally, this locus is filtered due to artifacts from multiple sequence alignment (MSA), as indicated by the green square.

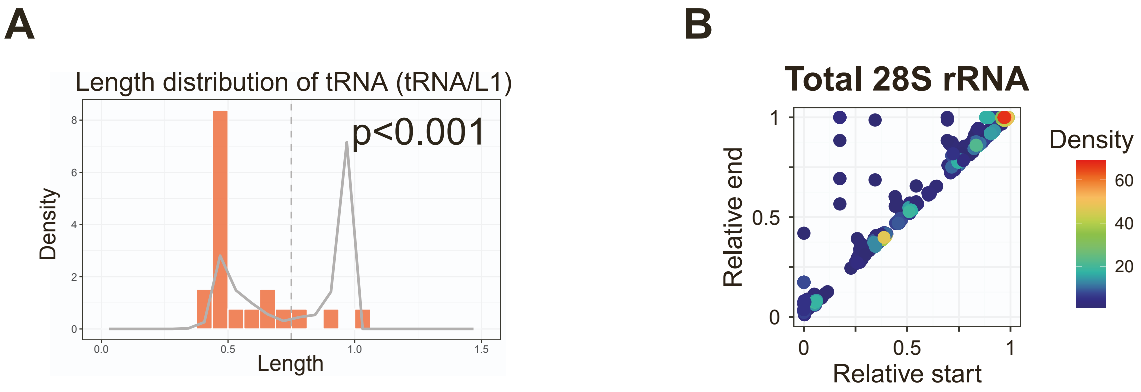

Figure S5. Characterization of newly identified RNA repeat associated LINE-1 chimeric insertions, Related to Figure 3. (A) Compared to the abundance of full-length tRNA in the genome generally, chimeric LINE-1 associated tRNAs are frequently truncated. This association was evaluated using Fisher's exact test where those <75% of the full tRNA are counted at truncated. (B) In the genome, most 28S rRNA pseudogenes retain sequence only at their 3′ end.

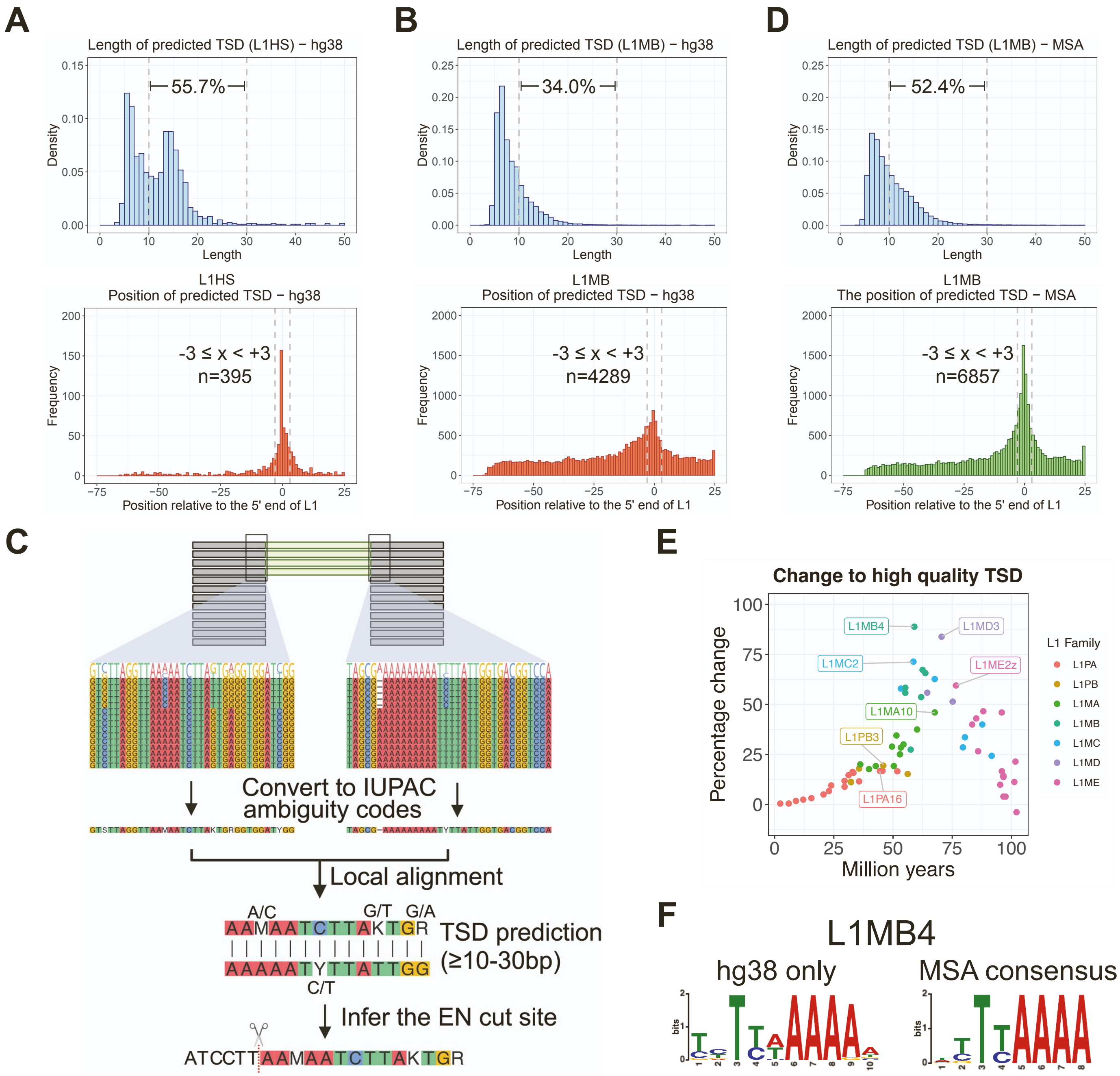

Figure S6. Predicting TSDs of LINE-1 from MSA, Related to Figure 3 and 4 (A) Upper panel: Distribution of predicted target site duplication (TSD) lengths for L1HS elements using the human reference genome hg38 only. The length of predicted TSDs shows two distinct peaks. The first peak, representing shorter lengths, indicates failed TSD predictions, while the second peak corresponds to expected TSD lengths (10-30 bp). Lower panel: Position of the predicted TSD relative to the annotated 5′ end of L1HS elements. The numbers indicate the frequency of events where the TSD is located within a 3 bp window upstream or downstream of the 5′ end of the LINE-1 insertion. (B) Upper panel: Distribution of predicted TSD lengths for L1MB elements using the human reference genome only. Lower panel: Position of the predicted TSD relative to the annotated 5′ end of L1MB elements. (C) Workflow for TSD Prediction: LINE-1 junction sequences are extracted and converted into IUPAC ambiguity codes to account for mutations in TSDs over time. These sequences are then aligned using local alignment methods to predict TSDs and EN motifs. (D) Upper panel: Distribution of predicted TSD lengths for L1MB elements using MSA cross genomes. Lower panel: Position of the predicted TSD relative to the annotated 5′ end of L1MB elements. (E) Percentage Improvement in high-quality TSD. High-quality TSDs are defined as those with a length of 10–30 bp and located within 3 bp upstream or downstream of the 5′ end of LINE-1. The percentage improvement in high-quality TSDs was calculated by comparing MSA-based predictions to predictions made exclusively using the hg38 reference genome. (F) Computed EN Motif for L1MB4 Elements: The EN motif of L1MB4 elements was predicted using MEME-suite with the sequences derived from hg38 and MSA-based approaches, demonstrating the improvement in TSD predictions for older LINE-1 families and allowing for EN motif prediction.

A

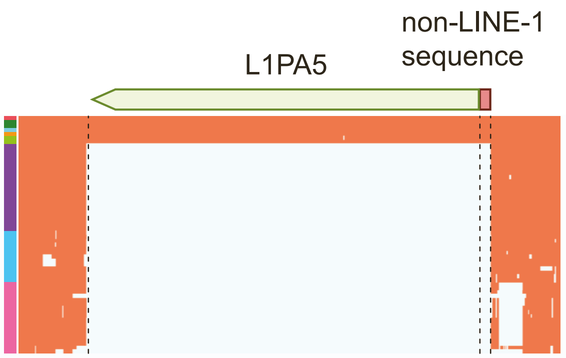

B

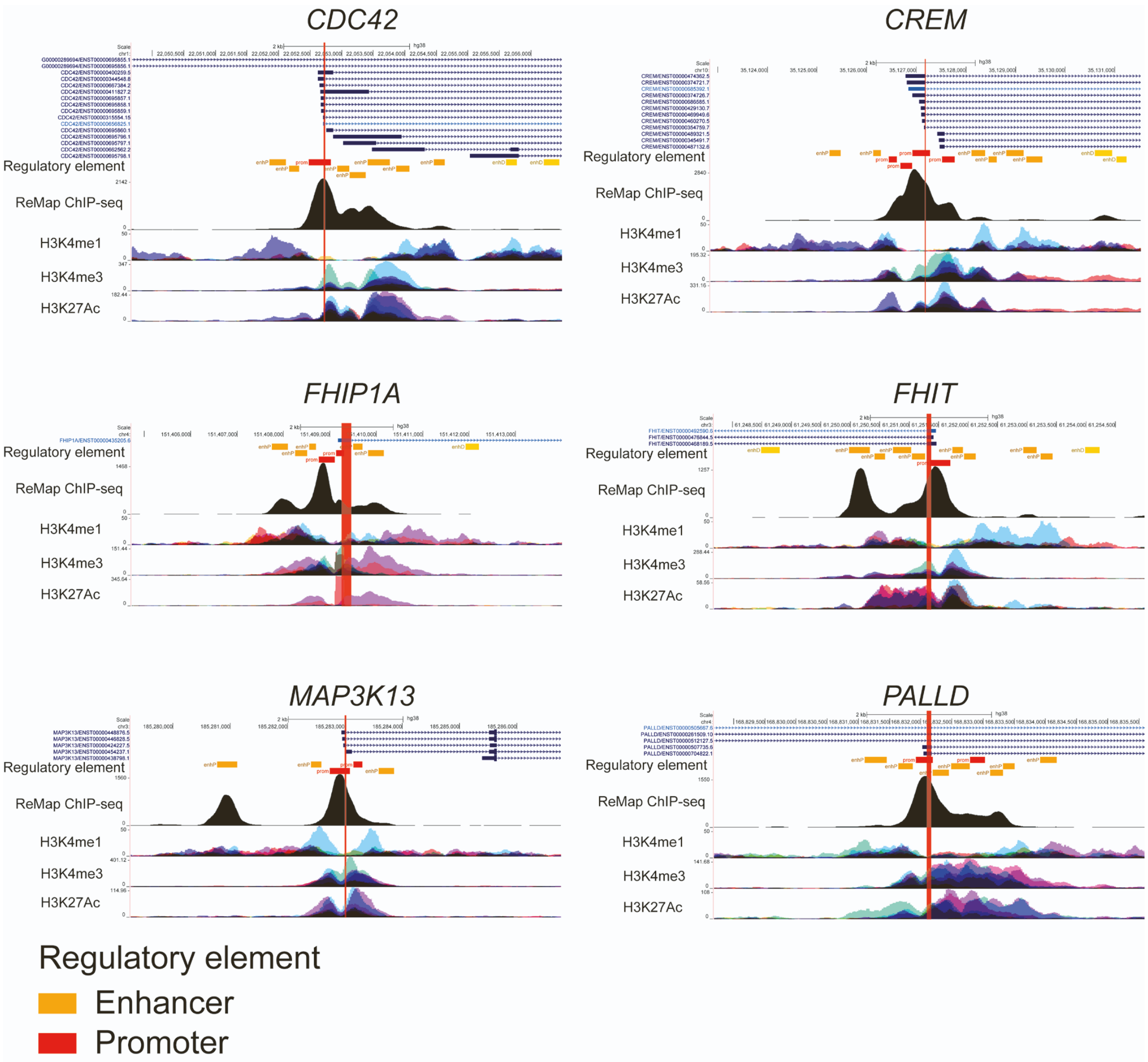

Figure S7. Regulatory elements overlapping with the first exon of mapped Genes, Related to Figure 5. (A) MSA showing a chimeric insertion composed of a LINE-1 element and non-LINE-1 sequence. (B) UCSC Genome Browser tracks showing regulatory elements overlapping the first exons of *CDC42*, *CREM*, *FHIP1A*, *FHIT*, *MAP3K13*, and *PALLD*. The portion of the first exon mapped to the chimeric insertion is highlighted in red. These first exons are associated with promoters enriched in transcription factor binding sites (ReMap ChIP-seq), regulatory regions and active histone marks, including H3K4me1, H3K4me3, and H3K27Ac, indicating active regulatory elements.

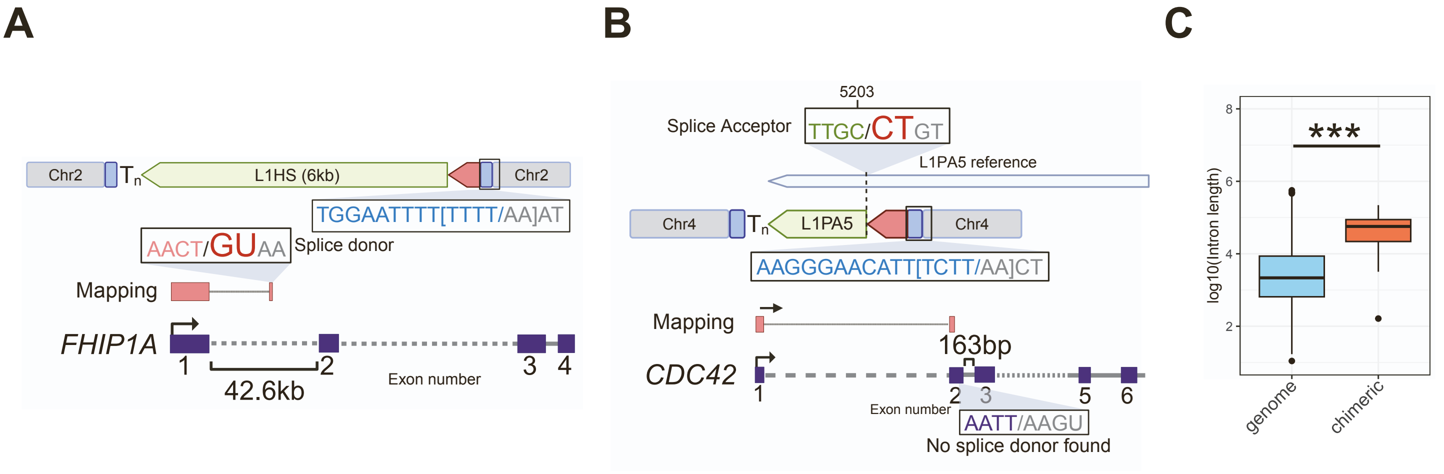

Figure S8. Examples of chimeric LINE-1 insertion with gene transcripts and related features, Related to Figure 5. (A) A chimeric LINE-1 insertion containing classic target site duplications (TSDs) at both ends, an endonuclease (EN) motif, and a poly(A) tail at the 3′ end. The upstream sequence of the LINE-1 element maps to an intron of *FHIP1A*, which contains a cryptic splice donor motif. The LINE-1 counterpart maps to the beginning of the L1HS reference sequence. (B) Another example of a chimeric LINE-1 insertion also contains classic TSDs at both ends, an EN motif, and a poly(A) tail at the 3′ end. The upstream sequence maps to the second exon of *CDC42*. However, no canonical splice donor motif is observed in this case. A splice acceptor motif is identified in the LINE-1 counterpart, upstream of position 5203 in a L1PA5 reference sequence. (C) The median length of the introns separating the last mapped exon, and the next exon is 48 kb, significantly longer than the common first and second intron length in the genome, which has median length of 2.2kb. The differences were statistically analyzed using the Wilcoxon's Rank Sum test, with *** denoting a p-value < 0.001.

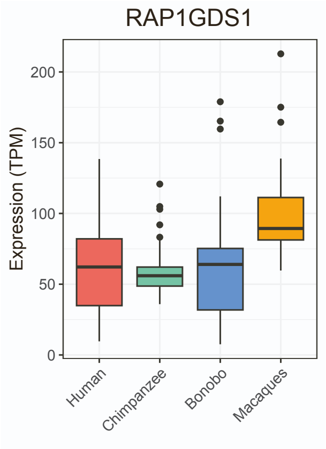

Figure S9. mRNA Expression levels of *RAP1GDS1* in the brain of primates, Related to Figure 6.

Active period	Group	Members
**1**	Human	L1HS
**1-3**	Primate	L1PA2
**3**		L1PA3
**4-5**		L1PA4
**5**		L1PA5
**6**		L1PA6
**6-7**		L1PA7, L1PA15-16
**7**		L1PA8, L1PA8A, L1PA10, L1PA11, L1PA12, L1PA13, L1PA14, L1PA15, L1PA16, L1PA17, L1PB1, L1PB2, L1PB3, L1PB4, L1MA1, L1MA2, L1MA3
**7-8**		L1MA4, L1MA4A, L1MA5, L1MA5A, L1MA6
**8-10**	Primates/Mammalian	L1MC1
**8-9**		L1MA7, L1MA8, L1MA9, L1MA10, L1MB1
**10**	Mammalian	L1MB2, L1MB3, L1MB4, L1MC2
**10-11**		L1MB5, L1MB7, L1MB8, L1MC3, L1MD1
**11**		L1MC4, L1MC4a, L1MC5, L1MC5a, L1MD2, L1MD3, L1ME1, L1ME2, L1ME2z, L1ME3, L1ME3A, L1ME3B, L1ME3C, L1ME3Cz, L1ME3D, L1ME3E, L1ME3F, L1ME3G, L1ME4a, L1ME4b, L1ME4c, L1ME5, HAL1, HAL1b, HAL1M8, HAL1ME
**11-12**		X9_LINE

Table S1. Predicted active period of LINE-1 families based on insertion timing, related to Figure 2. A LINE-1 family is considered active at a specific timepoint if ≥20% of its loci are inferred to have been inserted at that timepoint.
